# Interface frictional anisotropy of dilative sand

**DOI:** 10.1038/s41598-024-56621-1

**Published:** 2024-03-14

**Authors:** Muhammad Naqeeb Nawaz, Seung-Hun Lee, Song-Hun Chong, Taeseo Ku

**Affiliations:** 1 Department of Civil Engineering, Sunchon National University, 255 Jungang-ro, Sunchon-si, Jeollanam-do 57922 Republic of Korea; 2 Department of Civil and Environmental Engineering, Konkuk University, 120 Neungdong-ro, Gwangjin-gu Seoul, 05029 Republic of Korea

**Keywords:** Frictional anisotropy, Dilative sand, Direct shear apparatus, Cranial shearing, Caudal shearing, Interface friction angle, Interface dilation angle, Civil engineering, Engineering, Materials science, Structural materials

## Abstract

Understanding direction-dependent friction anisotropy is necessary to optimize interface shear resistance across soil-structure. Previous studies estimated interface frictional anisotropy quantitatively using contractive sands. However, no studies have explored how sand with a high dilative tendency around the structural surface affects the interface shear response. In this study, a series of interface direct shear tests are conducted with selected French standard sand and snakeskin-inspired surfaces under three vertical stresses (50, 100, and 200 kPa) and two shearing directions (cranial → caudal or caudal → cranial). First, the sand-sand test observes a higher dilative response, and a significant difference between the peak and residual friction angles (ϕ_peak_ − ϕ_res_ = 8°) is obtained at even a lower initial relative density D_r_ = 40%. In addition, the interface test results show that (1) shearing against the scales (cranial shearing) mobilizes a larger shear resistance and produces a dilative response than shearing along the scales (caudal shearing), (2) a higher scale height or shorter scale length exhibits a higher dilative tendency and produces a higher interface friction angle, and (3) the interface anisotropy response is more pronounced during cranial shearing in all cases. Further analysis reveals that the interface friction angle and dilation angle are decreased with the scale geometry ratio (L/H). For L/H values between 16.67 and 60, the interface dilation angle varies between 9° and 4° for cranial first shearing and 3.9°–2.6° for caudal first shearing. However, the difference in dilation angle within the same shearing direction is less than 1°.

## Introduction

Frictional anisotropy is mainly controlled by loading direction and the relative surface roughness between structural material and surrounding soil types^1–4^. Various types of textured surfaces have been developed to analyze the effect of surface roughness on interface frictional anisotropy^5–9^. Recently, bio-inspired systems have led to new developments in engineering^10,11^. One such system that has received significant attention is the snakeskin, which can mobilize direction-dependent frictional resistance while moving over ventral scales. Those ventral scales induce lower frictional resistance during the movement of a snake in the forward direction (caudal movement), and higher frictional resistance in the backward direction (cranial movement)^12,13^.

Inspired by the frictional characteristics of snakeskin, many studies have replicated the ventral scale geometry to achieve the desired frictional resistance during soil-structure interactions in various geotechnical engineering applications^5,14–19^. The frictional anisotropy induced by the bio-inspired surfaces was qualitatively analyzed using a conventional direct shear apparatus under a single vertical stress of 75 kPa^5^. The effect of bio-inspired surface patterns on interface frictional anisotropy was analyzed during monotonic loading conditions^8^. During the pullout and installation in sands, bio-inspired piles exhibited that the shaft capacity varies depending on the direction of load transfer^20^. The bio-inspired piles exhibited greater skin friction when subjected to tensile pullout compared to when they were jacked^21^. The bio-inspired pile pulled out in the cranial direction produces less vertical displacement and higher skin friction resistance than the caudal direction with smooth surface pile^19^. In addition, other studies explored the interfacial shear behavior between various sands and bio-inspired surfaces during the two-way interface direct shear tests^17,22^. Those sands showed pronounced contractive tendencies due to more rounded particle shape, and dependency of interface behavior on stress history. In the two-way shearing interface tests, cranial shearing direction produced higher peak shear stresses and dilation than caudal shearing. In the same shearing direction, a larger shearing resistance was mobilized in the second shearing cycle than in the first cycle. This was because contractive sands tended to compress and densify upon second shearing cycle. However, there is the need to understand how sand with higher dilative tendency around structural surface has effect on the interface shear behavior of sand.

This study aims to quantify loading direction-dependent frictional anisotropy using dilative sand. A modified direct shear apparatus is adopted to accurately evaluate the interface shear behavior for the application of nearly constant vertical stress during the shearing process. French standard sand (CEN Standard Sand—EN 196-1) categorized into three particle ranges is used to produce dilative response. A series of interface direct shear tests are performed using six snakeskin-inspired surfaces and one untextured surface under three initial vertical stresses (50, 100, and 200 kPa) and two-way shearing directions (cranial → caudal or caudal → cranial). The results are analyzed to better understand how the interface scale geometry formed with dilative sand has a quantitative effect on the interface shear response and frictional anisotropy. The discussion summarizes the interface friction angle and dilation angle as functions of the scale geometry ratio.

## Experimental materials and methods

### Textured surfaces and sand properties

This study uses six snakeskin-inspired surfaces made of polycarbonate material. Each surface is comprised of a 72 mm textured central part and a 45 mm untextured part on both sides to ensure enough distance from the sidewalls of the shear box [more details refer to^22^]. The different combinations of scale geometries are listed in Table 1.

**Table 1:** Geometrical parameters of bio-inspired surfaces: (a) characteristics of surfaces tested in this study; (b) illustration of a snakeskin-inspired surface and two-way shearing.

No	Type	Scale length, L [mm]	Scale height, H [mm]	N [ ]
(a)				
1	Textured	6	0.3	12
2		12	0.1	6
3		12	0.3	6
4		12	0.72	6
5		18	0.3	4
6		24	0.3	3
7	Untextured	–	–	–
(b)				


N indicates the number of surface scales. The total length of surface scales is fixed as 72 mm in all plates.

**Figure 1:**
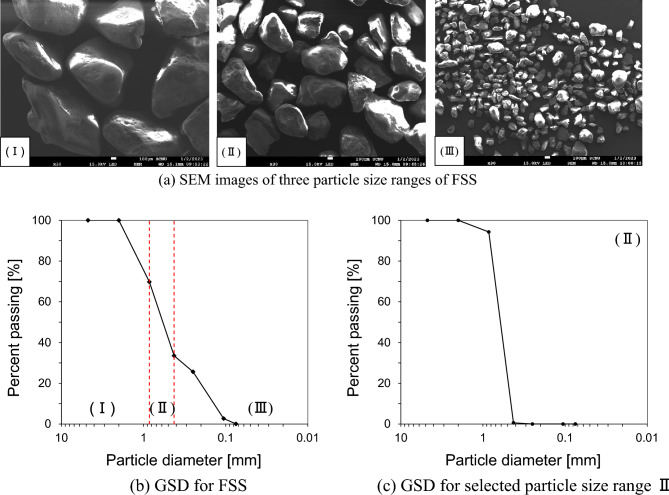
The particle size characterization of French standard sand (FSS): (**a**) SEM images of three particle size ranges of FSS; (**b**) Grain size distribution curve (GSD) of FSS; (**c**) Grain size distribution curve (GSD) of selected particle size range II. Based on the SEM image II, the shape of selected particles can be classified as angular, and the grain size distribution curve shows a uniform particle size range with C_u_ close to 1.44 (refer to Table 2).

In this study, French standard sand (FSS) is selected, a commonly used material for manufacturing cement mortar. It is composed of different particle sizes (Fig. 1), and can be categorized into three particle ranges (4.75–0.85 mm, 0.85–0.425 mm, and 0.425–0.075 mm). The preliminary interfacial tests show that (1) larger particles in the 4.75–0.85 mm size range (I) damage the snakeskin-inspired solid surfaces or breaks particles; (2) smaller particles in the 0.425–0.075 mm size range (III) intervene into either the LM (Linear Motion) guide rail or the small opening that exists on between the upper and lower shear box. Therefore, the 0.85–0.425 mm sized particles (II) are selected. SEM image for particles in 0.85–0.425 mm size range reveals angular shaped morphology of sand grains [Fig. 1(a)]. Generally, angular shape soil particles have higher difference between maximum void ratio (e_max_) and minimum void ratio (e_min_)^23^. However, this difference is relatively small (e_max_ − e_min_ = 0.19) for the selected FSS particles. The grain size distribution curve of selected soil particles confirms a uniform particle size with a coefficient of uniformity of 1.44 (Fig. 1c). The basic properties of the selected particle size range are listed in Table 2.

**Table 2:** Basic properties of selected French standard sand (FSS) used in this study.

Properties	Value
Coefficient of curvature C_c_ [ ]	0.93
Uniformity coefficient C_u_ [ ]	1.45
Average particle size D_50_ [mm]	0.61
Maximum void ratio e_max_ [ ]^a^	0.77
Minimum void ratio e_min_ [ ]^a^	0.58
Specific Gravity G_s_ [ ]	2.64
Friction angle ϕ_peak_ [°]^b^	39
Friction angle ϕ_residual_ [°]^b^	31
Dilation angle ψ [°]^b^	5.7

^a^The maximum and minimum void ratios are measured according to^24,25^.

^b^Direct shear test using a 2.5-inch circular shear box. The dilation angle is determined using Taylor^’^s flow rule^26^ at single vertical stress of 100 kPa.

### Modified direct shear test

**Figure 2:**
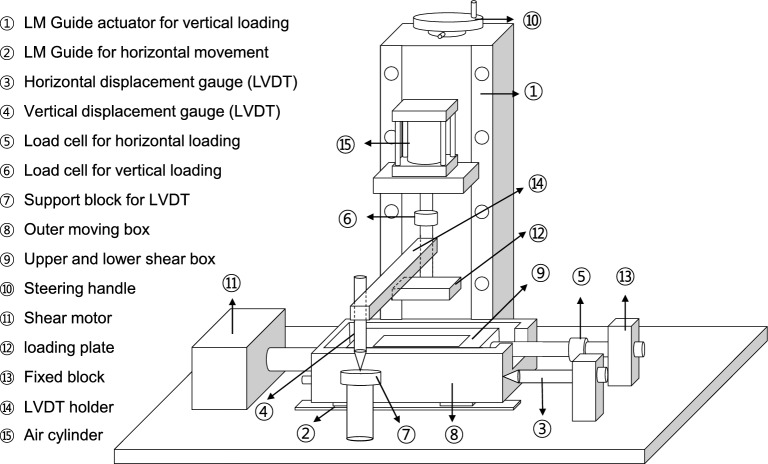
Schematic of a modified direct shear apparatus modified from^22,34^. For the interface tests and to accommodate snakeskin-inspired surfaces, the vertical stress application through the loading rod mounted on a steel ball and rectangular loading plate in the conventional apparatus is replaced by a rectangular loading plate attached to a vertical loading frame. For soil-soil tests, a circular shear box with a circular loading plate fixed to the vertical loading rod is used. Note that the loading plate and loading rod were not fixed in the conventional apparatus.

Direct shear test apparatus is widely used for determining soil shear strength parameters^27–29^. However, it has limitations including tilting of the upper loading plate and rotation of the upper shear box. Those potential problems eventually hinder quantifying shear response^30–33^. Previous studies modified the conventional direct shear apparatus for soil-snakeskin-inspired surface tests and soil-soil tests^22,34^. Figure 2 presents a schematic diagram of the direct shear test apparatus. The main modifications are herein explained as follows:

*Loading Plate* The vertical stress applied through the loading rod mounted on a steel ball and loading plate in the conventional apparatus is replaced with a loading plate attached to a vertical loading frame. Note that the loading plate and rod were not fixed in the conventional apparatus.

*Shearing System* The linear motion guide (LM guide) is installed for smooth and frictionless movement of the lower shear box bolted to the outer moving box. The lower shear box is the platform over which the snakeskin-inspired surface is fastened to perform the interface tests.

*Loading System* The vertical loading system uses an air cylinder. Additionally, the pressure gauge is installed to monitor the applied air pressure. The shear motor is used to easily control the constant shear strain rate.

*Sensor System* The vertical and horizontal loads applied to the specimen are measured using each load cell. In particular, a reaction arm is installed to connect the fixed block with the upper shear box. It facilitates transferring the friction force generated from the soil–plate interface to a horizontal load cell. Two LVDT sensors are installed to measure vertical and horizontal displacements.

### Experimental methods

A total of 39 interface direct shear tests are performed on six snakeskin-inspired surfaces and one untextured surface under three initial vertical stresses (50, 100, and 200 kPa) and two-way shearing directions. The two-way shearing consists of (1) cranial → caudal test: cranial shearing direction during the first half cycle and then caudal shearing during the second half cycle and (2) caudal → cranial: caudal direction during the first half cycle and cranial shearing during the second half cycle (refer Table 1b).

Sand specimen is air-pluviated over the snakeskin-inspired surface in the shear box for targeting an initial relative density of 40%. Based on the 10% shear strain failure criterion, the sand specimen is sheared until a displacement of 6 mm in a circular shear box for the sand-sand test and of 10 mm for the sand-snakeskin inspired interface tests under a constant 1 mm/min shear rate. All sensors are connected to a data logger to record and save data automatically. Also, the LabView program is used for the initial setting of sensors and continuous monitoring. The shear stress is calculated by dividing the measured shear force by the cross-sectional area of the specimen. All test results including the interface friction (ϕ_peak_) and dilation angle (ψ) are summarized in Table. 3.

**Table 3:** Summary of interface friction strength obtained from modified direct shear apparatus with various scale geometries, three vertical stresses, and two-way shearing: (a) Cranial → Caudal; (b) Caudal → Cranial.

L [mm]	H [mm]	(a) Cranial → Caudal									
		Cranial first shearing					Caudal second shearing				
		σ′_v_ [kPa]	τ_peak_ [kPa]	ϕ_peak_ [°]	ϕ_res_ [°]	Ψ [°]	σ′_v_ [kPa]	τ_peak_ [kPa]	ϕ_peak_ [°]	ϕ_res_ [°]	Ψ [°]
6	0.3	50	62.3	43.4	41.2	–	50	24.7	24.5	22.9	–
		100	100.1			7.4	100	52.1			3.6
		200	183.0			–	200	87.4			–
12	0.3	50	52.7	39.2	36.4	–	50	27.2	23.8	22.4	–
		100	89.6			6.0	100	51.1			3.2
		200	156.2			–	200	83.5			–
18	0.3	50	39.5	32.5	30.5	–	50	25.8	22.8	21	–
		100	69.6			4.0	100	44.0			2.6
		200	122.9			–	200	81.9			–
24	0.3	50	41.9	31.7	–	–	50	23.5	21.5	–	–
		100	68.2			–	100	40.8			–
		200	117.4			–	200	76.9			-
12	0.1	50	40.2	27.0	–	–	50	22.2	20.4	–	–
		100	52.8			–	100	38.9			–
		200	97.4			–	200	72.8			–
12	0.72	50	67.3	46.7	45	–	50	29.2	26.0	24	–
		100	119.9			8.8	100	57.9			4.0
		200	202.4			–	200	92.1			–

L [mm]	H [mm]	(b) Caudal → Cranial									
		Caudal first shearing					Cranial second shearing				
		σ′_v_ [kPa]	τ_peak_ [kPa]	ϕ_peak_ [°]	ϕ_res_ [°]	Ψ [°]	σ′_v_ [kPa]	τ_peak_ [kPa]	ϕ_peak_ [°]	ϕ_res_ [°]	Ψ [°]
6	0.3	50	26.9	24.3	21.7	–	50	58.1	43.1	40.1	–
		100	49.5			3.5	100	105.7			7.2
		200	86.9			–	200	178.5			–
12	0.3	50	24.4	22.9	19.7	–	50	49.9	38.3	36.5	–
		100	46.6			3.4	100	84.6			5.3
		200	81.2			–	200	152.7			–
18	0.3	50	26.8	22.6	18.4	–	50	45.3	33.1	30.2	–
		100	43.6			2.8	100	76.5			3.7
		200	80.9			–	200	121.8			–
24	0.3	50	23.2	21.4	–	–	50	38.3	29.4	–	–
		100	39.5			–	100	68.8			–
		200	77.5			–	200	104.3			–
12	0.1	50	22.9	21.6	–	–	50	35.7	26.3	–	–
		100	41.8			–	100	51.6			–
		200	77.2			–	200	95.2			–
12	0.72	50	28.6	26.3	22	–	50	64.3	46.3	43.9	–
		100	53.1			3.8	100	118.8			7.4
		200	95.8			–	200	199.5			–
Untextured		50	16.5	18.4	–	–	–	–	–	–	–
		100	33.5			–	–	–			–
		200	66.4			–	–	–			–

The interface friction angles for varying scale geometries are quantified by selecting the peak shear stress at the corresponding vertical stress and shearing direction based on the Mohr–Coulomb failure criterion. The correlation coefficient R^2^ is above 0.98 in all the cases. The dilation angles are measured at 100 kPa vertical stress using Taylor’s flow rule^26^.

## Results and analysis

### Shear behavior of French standard sand

**Figure 3:**
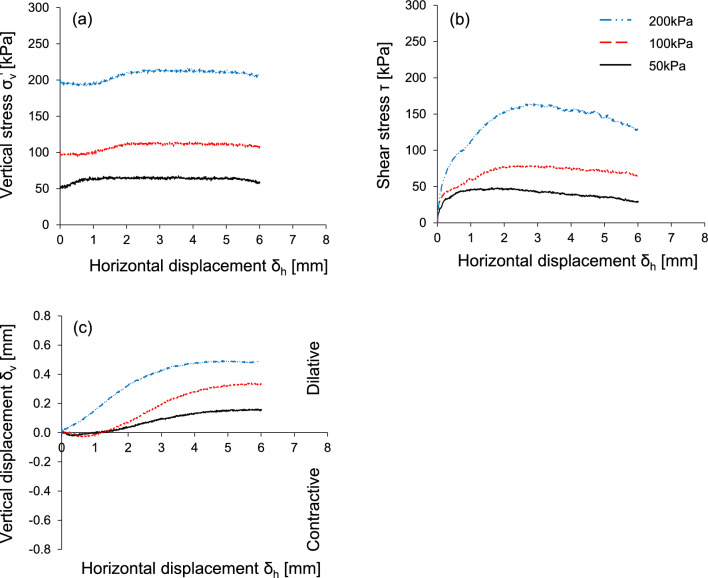
Soil-Soil shear response obtained from a modified circular direct shear apparatus under three different vertical stresses: Horizontal displacement against (**a**) vertical stress; (**b**) shear stress; (**c**) vertical displacement.

Figure 3 presents the soil-soil shear response under three initial vertical stresses (50, 100, and 200 kPa). The shear responses are plotted against the horizontal displacement related to the shear strain. During the initial shearing process, the change in the vertical stress follows the trend of vertical displacement associated with the volume change, yet it nearly remains constant after dilation (Fig. 3a). The shear stress-horizontal displacement response shows an increase in the initial stiffness (not shown here but can be indirectly assessed from the stress-displacement response) and peak shear resistance with the increase in vertical stress. The strain softening behavior is more pronounced at higher vertical stress. In addition, an increase in the vertical displacement occurs with an increase in the vertical stress. The volumetric dilatancy occurs after the initial contraction at 50 and 100 kPa (Fig. 3c). In summary, the dilative response is observed and a significant difference between the peak and residual friction angles (ϕ_peak_ – ϕ_res_ = 8°) is obtained at even a lower initial relative density D_r_ = 40%. These observations confirm the fact that the FSS particles can be classified as dilative sand.

### Interface shear behavior of snakeskin-inspired surfaces

**Figure 4:**
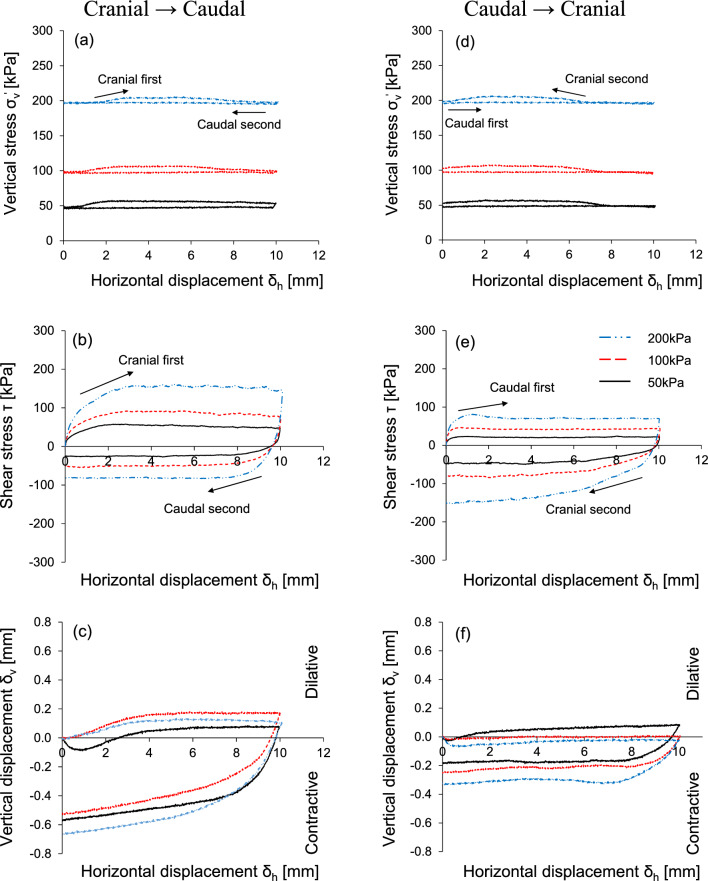
Response of interface frictional anisotropy using modified rectangular direct shear apparatus under three different vertical stresses: Horizontal displacement against (**a**,**d**) vertical stress; (**b**,**e**) shear stress; (**c**,**f**) vertical displacement. Experimental case is L = 12 mm and H = 0.3 mm.

Figure 4 shows the interface frictional anisotropy under three vertical stresses with the same scale geometry (L = 12 mm, H = 0.3 mm). Slight fluctuation of vertical stress takes place during cranial shearing process: the cranial shearing produces slight increase in the vertical stress while the caudal shearing keeps the vertical stress constant. This is because the interface shearing resistance during the cranial shearing process propagates toward the loading plate and eventually increases the local mean effective stress (Fig. 4a,d).

Figures 4b and e present shear stress–shear displacement response. Higher vertical stress produces larger shear stress, which is nearly similar for both the cranial → caudal and the caudal → cranial tests in the same shearing direction. While the cranial first shearing direction mobilizes higher shear resistance, the subsequent caudal shearing reduces shear stress. Conversely, caudal first shearing mobilizes lower shear resistance, and cranial second shearing enhances shear stress. Regardless of the starting direction, cranial shearing results in higher shear stress in all cases. A previous study^5^ explored the effects of shearing direction and scale geometry on induced soil deformation by analyzing particle image velocimetry. Shearing in the cranial direction enables the soil to latch onto the scales and increases the contact area of particles behind the scales. Eventually, the wedge-shaped shear failure mode tends to develop at the leading front of the scales, and shear localization propagates around the scales. However, the caudal shearing direction hinders the latching of the soil on the scales and fails to enhance the contact area. Accordingly, the induced failure deformation produces shear bands that are evenly propagated along the scale apex. As shown in Fig. 4c,f, the cranial first shearing process produces a more pronounced dilative tendency and volume change is larger for the cranial → caudal test than for the caudal → cranial test. This is because the shearing process in the cranial direction causes dilation of the soil at the leading end of the scales and contraction at the trailing end.

### Effect of scale geometry

**Figure 5:**
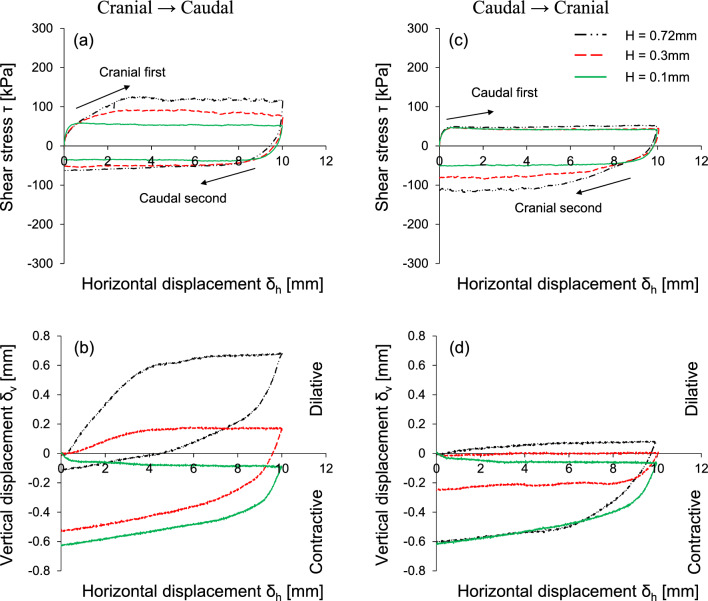
The effect of scale height on the interface shear response: Horizontal displacement against (**a**,**c**) shear stress; (**b**,**d**) vertical displacement. The applied vertical stress is 100 kPa and scale length L is fixed as 12 mm.

Figure 5 shows the results of the interface shear behavior by varying the scale height while keeping the scale length constant at 12 mm. A higher scale height mobilizes a larger shear stress in the cranial shearing direction, yet the difference of shear stress in the caudal shearing direction is less pronounced with scale height (Fig. 5a,c). The scale height changes the tendency of the vertical displacement associated with the volumetric response, as shown in Fig. 5b,d. For the cranial first shearing direction, a surface with a lower scale height (H = 0.1 mm) exhibits contractive behavior, whereas the responses at relatively higher scale heights (H > 0.1 mm) are dilative. The less dilatancy occurs for the caudal first shearing. This is because a higher height increases the interface roughness and develops larger individual passive wedges with the dilation of the soil at the front of the scale and the contraction of the soil behind the scale.

**Figure 6:**
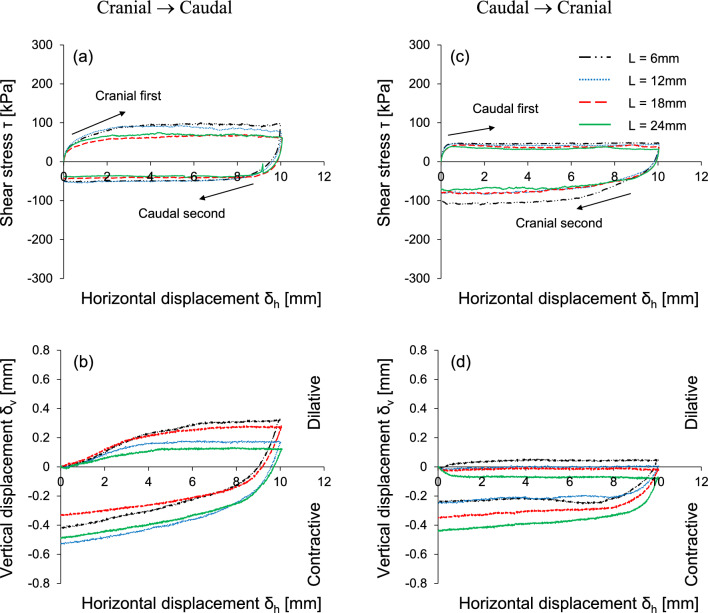
The effect of scale length on the interface shear response: Horizontal displacement against (**a**,**c**) Shear stress; (**b**,**d**) vertical displacement. The applied vertical stress is 100 kPa and scale height H is fixed as 0.3 mm. Note that a shorter scale length indicates a greater number of surface scales.

Figure 6 shows the response of the interface shear behavior by varying the scale length at the same scale height (H = 0.3 mm). Note that shorter scale length indicates more number of surface scales (but the total length remains the same). A shorter scale length mobilizes higher shear resistance during the cranial shearing direction, yet the caudal direction shows less change of shear stress with scale length (Fig. 6a,c). The dilative tendency is observed for all the scale lengths for the cranial → caudal test. But, contractive behavior is more pronounced for the caudal → cranial test except for the shorter scale length (L = 6 mm) as shown in Fig. 6b,d. This is because more textured surfaces (i.e., shorter scale lengths) mobilize more individual wedges, leading to a more dilative overall volumetric response during the cranial shearing process.

### Evolution of interface friction angle

**Figure 7:**
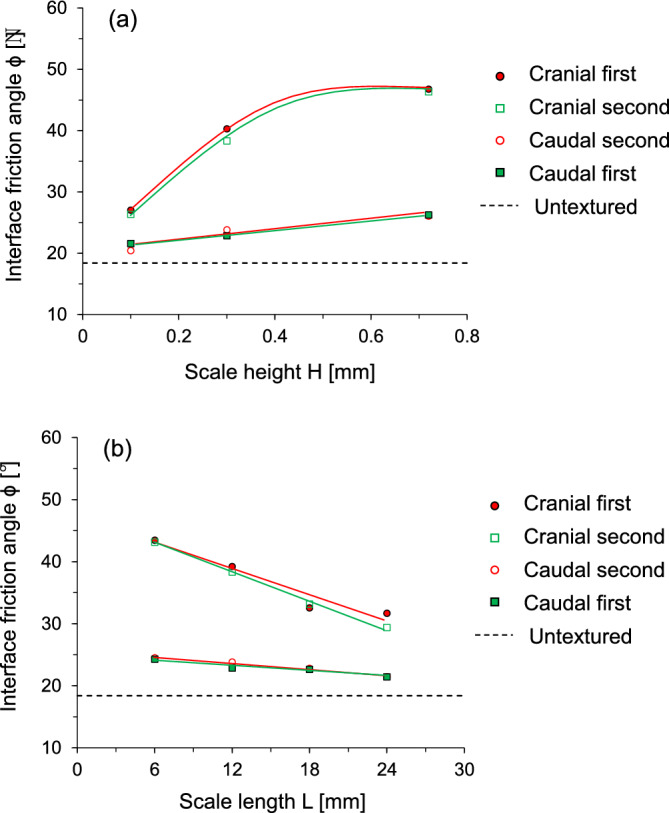
Interface friction angle as a function of snakeskin-inspired scale geometry under two shearing directions: (**a**) scale height (L = 12 mm fixed); (**b**) scale length (H = 0.3 mm fixed).

The interface friction angle is quantified to explore the effect of scale geometry and the shearing sequence. Figure 7a shows the evolution of the interface friction angle for snakeskin-inspired surfaces with varying scale heights at a constant scale length (L = 12 mm) under two shearing directions. Compared to the untextured surface, the textured surfaces produce higher interface frictional resistance in all cases. The interface friction angle is increased with scale height, and cranial shearing produces a higher interface friction angle than caudal shearing. For the cranial first shearing case, the difference between the friction angle at 0.1 mm and 0.72 mm scale heights is 19.7°, and for the cranial second case is 20°. A sharp increase in interface friction angle takes place within scale height between 0.1 and 0.3 mm during the cranial starting direction. Subsequently, an increase in height results in a moderate increase in the interface friction angle. A previous study explored the effect of surface roughness on sand-steel interface behavior^35^. The increase in the surface roughness related to the scale height produced a higher interface frictional strength, yet it reached an asymptotic value at a certain scale height. The trend of the interface frictional resistance is similar to the cranial shearing direction observed in this study. Meanwhile, the caudal shearing direction exhibits a slight increase in the interface friction angle, regardless of its sequence. The difference between the interface friction angles at 0.1 and 0.72 mm scale height is 4.7° and 5.6° for the caudal first and second cases, respectively.

Figure 7b shows the evolution of the interface friction angle for the snakeskin-inspired surfaces with varying scale lengths at a constant scale height (H = 0.3 mm) under two shearing directions. During cranial shearing, the interface friction angle dramatically decreases with increasing scale length. The differences between the friction angles at L = 6 mm and L = 24 mm scale lengths are 11.7° and 13.7° for the cranial first and second cases, respectively. Similarly, the difference in the interface angles is 3° for the first caudal case and 2.9° for the second caudal case. Cranial shearing direction produces higher interface friction angle regardless of the shearing sequence.

Indeed, the changing trend of interface friction angle is affected by the starting shear direction while the shearing sequence has no effect on the interface friction angle. Previous studies^17,22^ revealed that volumetric contraction at the trailing end of the scale during the starting shearing densified soil around the scale, difference in the interface friction angle between the cranial and caudal shearing directions was pronounced depending on the starting shearing direction, and thus the caudal first-caudal second shearing sequence produces larger friction difference than the cranial first-cranial second shearing sequence. However, the current study observes that the interface friction angle in the first shearing cycle is almost identical to that in the second shearing cycle, and thus densification effect during the first shearing is diminished. This is because angular particles during the first shearing cycle produce less volumetric contraction behind the scale, and the subsequent second shearing cycle does not cause a significant change in the mobilized shear resistance.

## Discussion and implications to geotechnical application

**Figure 8:**
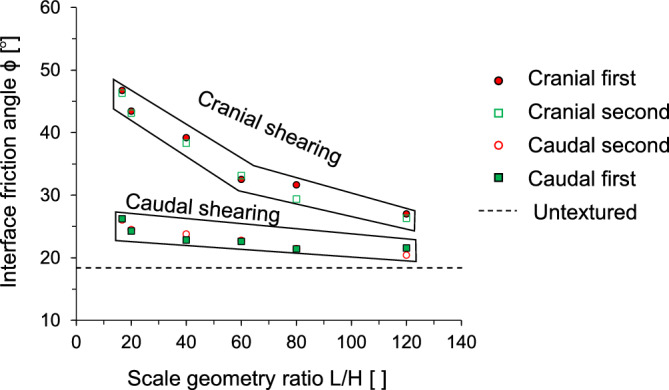
The interface friction angle as a function of normalized scale geometry L/H ratio. The interface friction angle significantly varies between cranial and caudal shearing directions at the given scale geometry ratio. However, the interface friction angle for similar shearing directions in a two-way shearing process (first cranial and second cranial, first caudal and second caudal) is almost identical.

The normalized scale geometry ratio, which is defined as the ratio of the scale length to height, is adopted to further analyze the interface frictional anisotropy. Figure 8 shows the interface friction angle as a function of the L/H ratio. Note that a large L/H indicates a lower scale height at the same scale length. Compared to caudal shearing, cranial shearing produces a higher interface friction angle at all scale geometry ratios regardless of the shearing sequence. In particular, the interface friction angle in the same shearing direction for the first shearing cycle (i.e., first cranial or first caudal) is almost identical to that for the second cycle of shearing (i.e., second cranial or second caudal) because of the unique dilative characteristics of the FSS particles, as discussed in the previous section. Interestingly, it is also observed that the shearing direction has a greater effect on the interface friction angle in regions with a lower L/H ratio.

Further analysis is conducted to investigate the evolution of the interface dilation angle across sand-bioinspired plate. It is selected for experimental cases of strain-softening response that show a significant distinction between the peak and residual shearing stresses for both the cranial → caudal and caudal → cranial tests. Then, the Taylor’s flow rule is applied as follows^26^.

1 $$\uppsi = \tan^{ - 1} \left( {\frac{{\uptau _{{{\text{peak}}}} }}{{{\upsigma ^ \prime }_{{\text{v}}} }} - \frac{{\uptau _{{{\text{res}}}} }}{{{\upsigma^ \prime }_{{\text{v}}} }}} \right)$$

where ψ is the interface dilation angle [°], τ_peak_ is the peak shear stress [kPa], τ_res_ is the residual shear stress [kPa], and σ′_v_ is the vertical stress [kPa].

**Figure 9:**
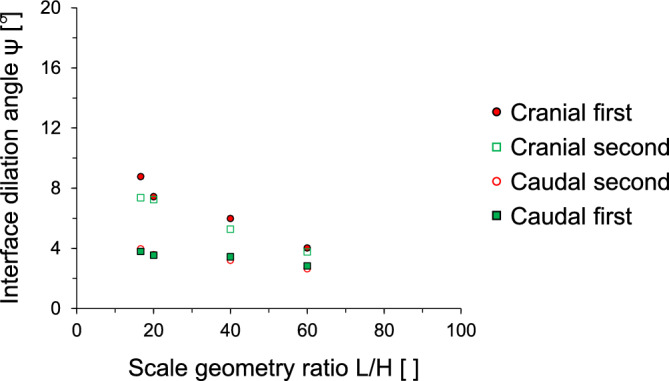
The dilation angle as a function of normalized scale geometry L/H ratio. The dilation angle is determined using the Taylor’s flow rule at 100 kPa vertical stress.

Figure 9 presents the interface dilation angles against different shearing directions and scale geometry ratios. The interface dilation angle decreases with the increase in the scale geometry ratio. For L/H values between 16.67 and 60, the interface dilation angle varies between 9°–4° for cranial first shearing and 3.9°–2.6° for caudal first shearing. Similar to the interface friction angle, the dilation angles for caudal shearing cycles are nearly identical regardless of the shearing sequence (i.e., first and second caudal). However, there is small difference (less than 1°) in the dilation angles for the cranial shearing cycles (first and second cranial). Indeed, this analysis confirms that the FSS dilates during the first shearing cycle and does not show further increase or changes in dilation during the second shearing cycle, which eventually leads to no considerable change in the mobilization of the dilation angle during the second shearing cycle. The stress-dilatancy equation was proposed by Bolton^36^. The empirical equation was initially developed based on tests conducted with dense sands under plain strain conditions. It can be expressed as follows:

2 $$\phi_{{{\text{peak}}}} = \phi_{{{\text{res}}}} + \, 0.{8}\psi$$

**Figure 10:**
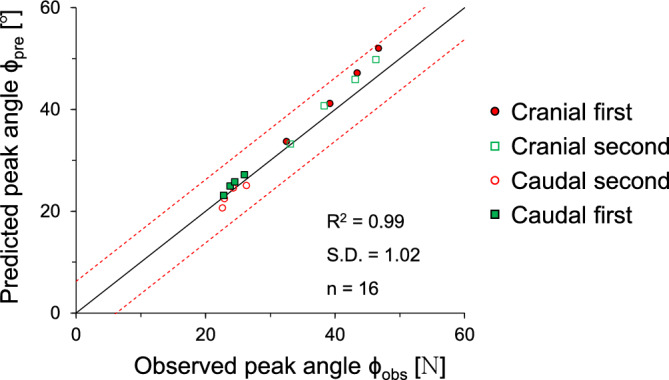
Comparison between the observed (this study) and the predicted interface friction angle. The empirical equation (ϕ_peak_ = ϕ_res_ + 0.8ψ) proposed by Bolton^36^ is used to predict the interface friction angle. Solid line shows the central trend defined by a 1:1 line. Dotted lines show ± 1 standard deviation from the central line.

Indeed, the difference between peak fiction angle and residual friction angle is related to a dilatancy coefficient. The empirical equation is re-evaluated by comparing with the results obtained from this study. Figure 10 shows a strong correlation between the predicted and observed peak interface friction angles. The observed peak interface friction angle exhibits high accuracy, with a substantial R^2^ value of 0.994, along with a low standard deviation (S.D).

The interface friction angle and dilation angle of dilative sand obtained from various scale geometries and different shearing directions help to understand the interface shear and dilation response, and ultimately optimal interface friction angle can be selected for the design of bioinspired geo-structures (e.g., driven piles, offshore onopoles, soil anchors, and tunnel boring machines) to achieve desired frictional anisotropy. For example, if the surface of a driven pile is textured with snakeskin-inspired scales having relatively higher L/H scale ratio, the minimized mobilized shear resistance could be achieved during the installation. The outcome of this study is not limited to snakeskin-inspired surfaces but is also applicable to other geotechnical infrastructure related interface shearing systems such as textured geomembranes and ribbed soil reinforcements. In this study, a lower initial relative density (D_r_ = 40%) is selected. However, note that change in relative density influences shear behavior. At the given scale geometry, higher initial relative density produces higher interface friction resistance, provided there is no particle crushing or damage to the interface material. Future studies are required to conduct in-situ field testing of textured bioinspired surfaces, incorporating data monitoring, and assessing real-world conditions to ensure practical effectiveness in diverse applications.

## Conclusions

This study used a modified direct shear apparatus for two-way shearing (i.e., cranial → caudal and caudal → cranial tests) on French standard sand with dilative tendency, which is sheared against snakeskin-inspired surfaces to quantify interface frictional anisotropy. The main conclusions of this study are as follows:

- The sand-sand direct shear results obtained using a modified circular shear box apparatus demonstrate the highly dilative nature of the FSS particles. At the low relative density of 40%, the significant difference between peak and residual friction angles (ϕ_peak_—ϕ_res_ = 8°) confirms the dilative nature of FSS.
- For cranial shearing in the first direction, the low scale height (H = 0.1 mm) results in contractive behavior, while higher scale heights (H > 0.1 mm) exhibit dilative responses. A shorter scale length enhances shear resistance during cranial shearing, with less change in shear stress observed for caudal shearing. Dilative tendencies are observed for all scale lengths in cranial → caudal tests, but contractive behavior is more pronounced in caudal → cranial tests, except for shorter scale lengths (L = 6 mm).
- A larger height and shorter scale length lead to increased interface friction angles, irrespective of shearing direction. Specifically, cranial shearing against surfaces with scale heights between 0.1 and 0.3 mm results in a substantial increase in the interface friction angle, while the increase becomes moderate between 0.3 and 0.72 mm. In contrast, the variation in interface friction angle during caudal shearing is moderate. Furthermore, a longer scale length significantly reduces the interface friction angle during cranial shearing and moderately during caudal shearing.
- The changing trend of interface friction angle with the scale geometry ratio L/H is affected by the starting shear direction while the shearing sequence has no effect on the interface friction angle. The interface friction angle in the first shearing cycle is almost identical to that in the second shearing cycle. This is because angular particles during the first shearing cycle produce less volumetric contraction behind the scale, and the subsequent second shearing cycle does not cause a significant change in the mobilized shear resistance.
- The interface dilation angle decreases with the increase in the scale geometry ratio. The dilation angles for caudal shearing cycles remain nearly identical regardless of the sequence (first or second), with only a minor difference (less than 1°) observed in the dilation angles for cranial shearing cycles (first and second). This analysis confirms that the FSS dilates during the first shearing cycle and does not show further increase or changes in dilation during the second shearing cycle.

## Data Availability

The data used is included in this manuscript.
